# Correlation Between Dysbiosis and Atrial Fibrillation: What’s New?

**DOI:** 10.3390/ijms27010073

**Published:** 2025-12-21

**Authors:** Beatrice Marinacci, Noemi Mencarelli, Giorgia Stornelli, Benedetta Pellegrini, Amedeo Amedei, Marialucia Gallorini, Rossella Grande

**Affiliations:** 1 Department of Pharmacy, “G. d’Annunzio” University of Chieti-Pescara, 66100 Chieti, Italy; beatrice.marinacci@unich.it (B.M.); noemi.mencarelli@phd.unich.it (N.M.); giorgia.stornelli@phd.unich.it (G.S.); benedetta.pellegrini@unich.it (B.P.); marialucia.gallorini@unich.it (M.G.); 2 Department of Innovative Technologies in Medicine & Dentistry, “G. d’Annunzio” University of Chieti-Pescara, 66100 Chieti, Italy; 3 Department of Experimental and Clinical Medicine, University of Florence, 50134 Florence, Italy; amedeo.amedei@unifi.it; 4 Network of Immunity in Infection, Malignancy and Autoimmunity (NIIMA), Universal Scientific Education and Research Network (USERN), 50139 Florence, Italy; 5 Laboratorio Congiunto MIA-LAB (Microbiome-Immunity Axis Research for a Circular Health), University of Florence, 50134 Florence, Italy

**Keywords:** gut microbiota, atrial fibrillation, dysbiosis, arrhythmias, cardiovascular disease, metabolites, *Ruminococcus*, *Bacteroides*, TMAO

## Abstract

Atrial fibrillation (AF), one of the most prevalent and widespread arrhythmias, has remained a heavy global burden in the past decade and directly influences the quality of human life. It is estimated that AF affects 2–4% of the world’s adult population, and it is predicted to double due to (i) life expectancy increasing and (ii) the rising frequency of undiagnosed cases. Recent studies suggest that the alteration of gut microbiota (GM), known as dysbiosis, could increase the AF risk. Since dysbiosis is a modifiable risk factor, interventions aimed at restabilizing gut eubiosis by diet, probiotics, or prebiotic supplements may represent a promising strategy for AF prevention and/or treatment, although current clinical evidence remains limited. In this scenario, it is very critical to understand which types of GM alterations or metabolites may be involved in the development of atrial AF, as this could open new strategies for managing the pathology. However, many gaps still need to be filled, as it remains unclear how dysbiosis contributes to AF across different populations and no fine characterization of the underlying pathogenic mechanisms is available yet. This review summarizes the last evidence on the association between GM dysbiosis and AF, highlighting the main proposed mechanisms, key microbial genes and metabolites involved, clinical implications and the need for further prospective studies.

## 1. Introduction

Cardiovascular diseases (CVDs) are the leading cause of morbidity and mortality worldwide, accounting for nearly one-third of all global deaths. They include a wide range of disorders affecting the heart and blood vessels, such as coronary artery disease, cerebrovascular disease, heart failure, peripheral arterial disease, and cardiac arrhythmias [1]. Among these, atrial fibrillation (AF) is the most common sustained cardiac arrhythmia and a major contributor to cardiovascular morbidity, mortality, and healthcare burden [2].

AF is associated with an increased risk of stroke, heart failure, and all-cause mortality; in addition, several recognized risk factors for AF (including age, sex, hypertension, obesity, and ischemic heart disease) have been shown to correlate with marked alterations in gut microbiota (GM) composition and function. Evidence from multiple observational cohort studies documents that disturbances in GM balance may play a role in AF development. These investigations have compared the GM profiles of AF patients and healthy controls, as well as among individuals with different AF subtypes (such as persistent or paroxysmal forms) and varying disease durations. Overall, the data support a relationship between gut microbiota and both the AF onset and progression [3].

In this scenario, a rapidly emerging area of interest is the heart–gut axis, which highlights the role of the intestinal microbiota in modulating the risk and progression of AF. The gut microbiota, through the production of bioactive metabolites and its interaction with the immune system, can directly influence the arrhythmogenic substrate [4]. Intestinal dysbiosis has been associated with increased gut permeability and systemic translocation of endotoxins such as lipopolysaccharide (LPS), which triggers chronic low-grade inflammation through Toll-like receptor activation, promoting atrial fibroblast activation, extracellular matrix deposition, and structural remodeling. Microbial-derived metabolites, including trimethylamine-N-oxide (TMAO), have been implicated in myocardial fibrosis and endothelial dysfunction, thereby contributing to the stabilization of arrhythmia [5]. Moreover, the microbiota may modulate autonomic tone via the gut–brain axis, influencing both vagal and sympathetic activity, key factors in AF pathogenesis. This autonomic imbalance synergizes with structural remodeling and ion-channel dysregulation to promote electrical instability, shortening of atrial refractory periods, and increased AF susceptibility. Recent human studies further support the mechanistic link between GM and AF. Patients with AF exhibit consistent alterations in microbial composition, including increased abundance of genera such as *Dialister*, *Dorea*, *Haemophilus*, *Klebsiella*, *Ruminococcus*, and *Veillonella*, and decreased levels of potentially protective genera such as *Butyricicoccus*, *Hungatella*, and *Prevotella* [6]. Elevated preoperative TMAO levels have been independently associated with postoperative AF, along with increased atrial fibrotic markers, including collagen I, collagen III, and fibronectin, suggesting that dysbiosis-driven metabolites can promote a fibrotic and pro-arrhythmic atrial substrate [7]. These findings open new therapeutic perspectives based on GM modulation, including dietary interventions, probiotics, prebiotics, and more advanced strategies with the aim of disrupting the vicious cycle linking inflammation, remodeling, and arrhythmogenesis [7].

## 2. Methods

The manuscript’s aim was to summarize and critically discuss the current evidence correlating GM dysbiosis to atrial fibrillation. This review was conducted by examining the literature available in English on PubMed, Scopus, and Web of Science (WOS), without any time restrictions. An electronic search was performed to identify suitable studies using the following keywords: “gut microbiota” and “atrial fibrillation”, “dysbiosis” and “atrial fibrillation”, sorted by best match on PubMed, classified by “Article title, Abstract, Keywords” on Scopus, and ordered by “Relevance” on WOS. All studies with correlations between the gut dysbiosis and atrial fibrillation were included, and the articles not relevant to the aim of the review, reviews, and case reports were excluded. Three reviewers independently extracted data from all the included studies using a predesigned extraction form (Microsoft Excel 2020, Microsoft Corporation, Redmond, WA, USA) for data collection and descriptive analysis, and any disagreement was solved by discussion. The main data from the selected studies were extracted and synthesized in narrative form.

A total of 279 manuscripts were found with these keywords, but only 21 studies fulfilled the selection criteria of the present mini-review and were included in analyses.

These studies showed a heterogeneous design:

- Nine [8,9,10,11,12,13,14,15,16] were observational cross-sectional studies;
- Two [17,18] were observational cross-sectional comparative studies;
- One [19] was an observational, cross-sectional, and in vivo study;
- One [4] was an observational cross-sectional and short-term longitudinal study;
- Three [2,3,20] were observational cohort studies with Mendelian randomization analysis;
- One [21] was a prospective observational cohort study;
- Two [22,23] were preclinical experimental studies;
- One [24] was a prospective observational study;
- One [25] was an observational cross-sectional study combined with in vitro and in vivo experiments.

The results were narratively synthesized, highlighting GM alterations, methodological differences, study outcomes, key microbial genes and metabolites identified, proposed mechanism, and clinical implications.

## 3. Pathophysiology of Atrial Fibrillation

Cardiac arrhythmias are disorders of heart rhythm that arise from alterations in impulse generation or conduction within the cardiac electrical system. Their clinical spectrum ranges from benign premature beats to life-threatening conditions such as ventricular fibrillation. The global prevalence of arrhythmias is significant, with atrial fibrillation representing the most common sustained arrhythmia and a major cause of morbidity and mortality worldwide [26]. AF is associated with an increased risk of thromboembolic events, heart failure, and decreased quality of life.

**Figure 1:**
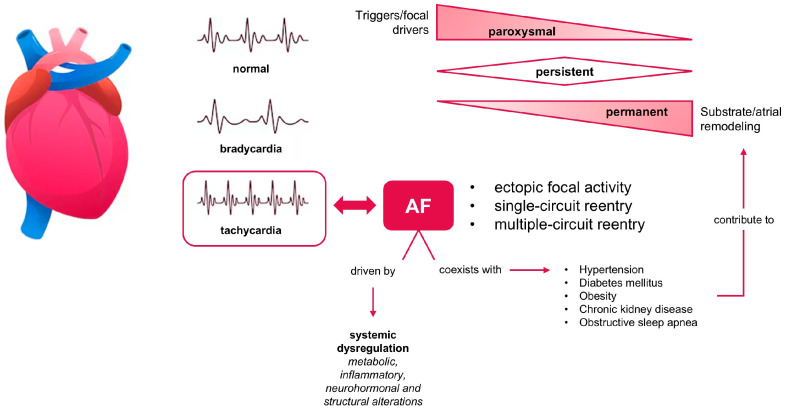
AF emerges from focal and reentrant mechanisms, driven by systemic dysregulation and comorbid conditions. Its progression, from paroxysmal to permanent, is shaped by evolving triggers and atrial remodeling.

The pathophysiology of arrhythmias is complex and multifactorial, involving structural, electrical, contractile, and autonomic remodeling. Atrial fibrillation commonly begins with paroxysmal episodes, characterized by spontaneous termination within seven days of onset [27]. When episodes fail to resolve spontaneously and require either pharmacological treatment or direct-current cardioversion, the condition is classified as pers+ùùistent AF. In the permanent form, restoration of sinus rhythm is no longer achievable or is considered clinically inappropriate [28]. Clinical and experimental evidence suggests that AF often follows a progressive course, evolving from paroxysmal to persistent and eventually to permanent stages. This progression is largely driven by atrial remodeling, a process promoted both by the arrhythmia itself and by underlying structural heart disease. In the early phases, electrical remodeling, primarily related to changes in the expression and function of cardiac ion channels, alters atrial electrophysiology and promotes conditions that facilitate AF maintenance. These alterations are largely reversible in the early stages following successful AF end, a phenomenon referred to as reverse remodeling (Figure 1). However, with advancing atrial pathology, remodeling becomes dominated by irreversible structural changes such as fibrosis and atrial dilatation, ultimately stabilizing the arrhythmia in its permanent form [29].

In detail, fibrosis, one of the major forms of arrhythmogenic remodeling, plays a central role in disease onset and progression. Different types of fibrotic changes can be observed: reactive interstitial fibrosis, which separates muscle bundles and disrupts their coordinated contraction, and reparative fibrosis that replaces necrotic cardiomyocytes and further compromises tissue integrity [28].

The autonomic nervous system contributes to AF, with imbalances in sympathetic and parasympathetic activity promoting arrhythmia maintenance and interacting with structural and inflammatory remodeling [30]. Beyond its electrophysiological manifestations, AF is increasingly recognized as a complex, multifaceted syndrome rather than a simple arrhythmia. This change in perspective comes from the understanding that AF arises from and contributes to a broad spectrum of systemic pathophysiological processes. Rather than being confined to the atrial myocardium, AF is embedded within a network of interrelated mechanisms involving metabolic, inflammatory, neurohormonal, and structural alterations that extend beyond the heart [31]. AF often coexists with comorbidities such as hypertension, diabetes mellitus, obesity, chronic kidney disease, and obstructive sleep apnea, all of which contribute to a pro-arrhythmic substrate through systemic inflammation, oxidative stress, and autonomic imbalance. These clinical conditions not only predispose to AF onset but also accelerate its progression and complicate its management.

In this context, AF can be regarded both as a consequence and as a driver of systemic disease, creating a bidirectional relationship that perpetuates cardiovascular dysfunction [31]. Moreover, the concept of AF as a syndrome underscores the relevance of individualized and multidisciplinary approaches to treatment that are not limited to rhythm or rate control alone but address the underlying causes, targeting modifiable risk factors and systemic contributors. This includes lifestyle modification, weight management, glycemic control, and treatment of sleep-disordered breathing, all of which have demonstrated efficacy in reducing AF burden and improving outcomes [32]. In addition, recognizing AF as a systemic syndrome has implications for research, paving the way for more effective, personalized therapies that target the fundamental contributors to AF [32].

## 4. Gut Microbiota Dysbiosis

The gut microbiota is now recognized as a functional organ essential for human health [33]. At birth, the gut is sterile, but it is rapidly colonized by bacteria transferred from the mother, both vertically and horizontally, during delivery [34]. Its initial composition depends on factors such as the type of delivery, nutrition, antibiotic use, infections, changes in diet, environment, and genetics [35,36,37,38]. However, the gut microbiota is not a static entity but a dynamic ecosystem whose composition varies over time and in response to both exogenous and endogenous factors [34,39,40,41]. In a state of eubiosis, when the gut microbiota maintains a balanced and functional composition, it is crucial for the host’s health, the development of immunity, protection against pathogens, and metabolism [33,41]. In this state of equilibrium, the phyla Firmicutes and Bacteroidetes predominate, but their ratio can vary between individuals and be altered in different clinical conditions [42]. Conversely, dysbiosis, known as the loss of eubiosis, is associated with autoimmune, metabolic, oncological, and neurodegenerative diseases [39,43].

Among the exogenous factors that influence the composition of the GM, diet plays a fundamental role: a high-fat diet modifies microbial diversity, shifting the balance towards Firmicutes at the expense of Bacteroidetes; a fiber-rich diet promotes the growth of short-chain fatty acid (SCFA) producing bacteria that can promote metabolic processes such as lipogenesis and gluconeogenesis [44,45,46]. From this perspective, interventions based on the use of probiotics, prebiotics, and postbiotics are emerging as possible tools for modulating the microbiota and restoring eubiosis conditions, although their effects depend largely on the strain and clinical context [47]. Probiotics mainly act by strengthening the intestinal barrier and competing with pathogens, while prebiotics support the selective growth of beneficial bacteria such as *Bifidobacterium* and *Lactobacillus*. Postbiotics, which include microbial metabolites and structural components, are attracting growing interest due to their stability and safety compared to live microorganisms [48]. Studies conducted on *Limosilactobacillus reuteri* DSM 17938 have highlighted the antimicrobial potential of cell-free supernatants and extracellular vesicles (EVs), whose content may vary depending on the physiological state of the bacterium, suggesting a differentiated role in communication between microbe and host [49,50,51].

At the same time, the use of pharmaceutical products, especially antibiotics, can lead to profound changes in the GM structure, which can potentially compromise host health with an increased risk of infections and complications [46,52,53,54]. However, the relationship between drugs and microbiota can be defined as bidirectional since drugs can alter the microbiota, while the microbiota can also influence the efficacy and safety of drugs [55,56]. The complex interaction between microbiota and drugs has led to the emerging field of *pharmacomicrobiomics*, a new discipline that aims to personalize therapies based on individual microbial profiles [57].

As previously mentioned, among the endogenous factors, the immune system plays a pivotal role in maintaining a symbiotic balance with the microbiota through non-inflammatory tolerance mechanisms, such as the regulation of the intestinal mucosal barrier, the secretion of Immunoglobulin A (IgA) and antimicrobial proteins [58,59,60]. Conversely, oxidative stress conditions and intestinal inflammation can promote dysbiosis by inhibiting beneficial anaerobic bacteria and promoting the growth of opportunistic bacteria such as *Enterobacteriaceae*, *Salmonella*, and *Escherichia coli* [46,61]. It is now well known that the consequences of dysbiosis are most evident in gastrointestinal disease, but it warrants emphasis that they can extend systemically [62]. In detail, the altered production of microbial metabolites such as short-chain fatty acids (SCFAs), trimethylamine (TMA), and trimethylamine N-oxide (TMAO) has been associated with metabolic dysfunction, insulin resistance, atherosclerosis, and endothelial alterations [63,64,65,66]. In this regard, intestinal dysbiosis is now recognized as a potential emerging risk factor for cardiovascular diseases, including hypertension, atherosclerosis, heart failure, and, more recently, atrial fibrillation [67]. The interaction between the microbiota and cardiovascular system involves systemic inflammation, oxidative stress, modulation of vascular tone and tissue remodeling, suggesting that the microbiota may be a potential therapeutic target [62,68,69,70]. With regard to AF, the GM specifically contributes to its pathophysiology through the production of bioactive metabolites, including SCFA, TMAO, indoxyl sulfate, LPS, and bile acids, which are capable of modulating inflammation, oxidative stress, cardiac remodeling, and atrial electrical conduction. Experimental studies indicate that alterations in these metabolites, together with NLRP3 inflammasome activation and atrial fibrosis, increase susceptibility to AF, while targeted interventions on the microbiota can reduce the risk and intensity of arrhythmia [5,71,72].

In addition to the use of probiotics, prebiotics, and postbiotics, new approaches are emerging, such as personalized nutrition, targeted microbial therapies, and the development of new-generation probiotics based on commensal strains with specific metabolic or immunomodulatory properties [73,74,75]. One of the most innovative approaches is Fecal Transplantation (FMT), which is moving beyond infectious disease, with ongoing clinical trials exploring its effectiveness in metabolic syndrome, Inflammatory Bowel Disease (IBD), and even neuropsychiatric and neurodegenerative disorders [76,77,78]. However, despite promising advances, several challenges remain, including the need for standardized protocols, long-term safety data, and a deeper understanding of host–microbiota interactions. Ultimately, the integration of microbiome science into clinical practice could usher in a new era of personalized medicine, in which the gut microbiota becomes both a diagnostic tool and a therapeutic target [79].

## 5. Discussion

### 5.1. Evidence on Association Between Gut Dysbiosis and Atrial Fibrillation

As supported by a plethora of studies, the gut microbiota is considered a “hidden organ” due to the numerous host functions that support digestion, protection against pathogens, response to drugs, immune regulation, etc. [40,80]. It is well known that, starting from the very first days of life, the GM-host interaction is crucial for driving multiple physiological processes, such as shaping the brain structure or modulating the immune system [81,82,83]. At the same time, the GM alterations throughout life can have an impact on multiple organs, influencing host health. As described above, when dysbiosis occurs, it can lead to several pathological outcomes [39].

Recently, the term “gut–heart axis” has started to be used with regard to the strong connection between the gastrointestinal tract and the cardiovascular system [84]. It clearly explains the interplay of gut microbiota and the heart, suggesting the key role that microbes have in regulating cardiac physiology and function [85]. In Table 1, we summarized all the original articles that explored the correlation between gut dysbiosis and AF, highlighting the potential mechanisms involved. A common observation in all studies was that AF patients showed a dysbiotic gut microbiota and, unsurprisingly, the key features of this condition were comparable among the majority of the works. *Bacteroides* and *Ruminococcus* were two of the genera that were extensively reported as more abundant in AF patients [4,8,10,12,17,18,19,20,22]. Those belonging to the *Bacteroides* genus are part of the GM commensal, participating in nutrient metabolism and immune modulation [86]. Some studies have proved that certain *Bacteroides* can have protective properties; for example, *B. fragilis* and *B. vulgatus* showed to have anti-inflammatory effects in LPS-induced systemic inflammation in mice and in hyperlipidemia in rats [87,88]. In contrast with these findings, most of the studies selected in this review documented that a higher abundance of *Bacteroides* is a typical feature in AF patients, related to a pro-inflammatory environment. Considering the dysbiosis that occurs during AF, we can suppose that the transition to a “harmful phenotype” is correlated to the microbial imbalance, which disrupts the physiological interactions of the eubiotic environment [89]. Moreover, we can speculate that the effect is species-specific.

At the same time, *Ruminococcus* is a genus that may also be associated with inflammation. As reported by Hall et al. [90], a strong increase in *R. gnavus* in IBD patients could disrupt the mucus layer, potentially contributing to inflammatory outcomes. Similarly, Henke and colleagues confirmed that this species, associated with Crohn’s disease, can release a pro-inflammatory polysaccharide [91] while other authors detected an increase in *Eubacterium*, *Blautia*, and *Dorea* [8,20,24], as well as *Klebsiella*, *Streptococcus*, and *Citrobacter* in AF patients [4,24]. Additionally, some of them were able to identify altered bacteria at the species level, namely *Enterococcus faecium* [8], *Methanobrevibacter smithii* [20], *Escherichia coli* and *Eubacterium rectale* [10].

Regarding the genera decreased in AF condition, several studies highlighted a reduction in *Faecalibacterium* [13,20]; in detail, two of them reported a lower abundance of *F. praustnitzii* [8,10], a commensal bacterium of the human gut, which has gained increasing interest within the last decade. Indeed, it is one of the most represented butyrate-producing species, well known for its anti-inflammatory effects [92]. Due to its contribute in gut homeostasis, it has started to be credited with microbiota-regulating properties, being also regarded as a biomarker of overall health [93,94]. Therefore, the decreased abundance of this species may contribute to the loss of a protective mechanism against inflammation.

Another common AF feature is the lower abundance of *Prevotella*, a predominant genus in the human oral cavity but also represented within the gut. Interestingly, in eubiosis, the *Prevotella* spp. presence appears to be inversely correlated with *Bacteroides* [95] and this tendency is maintained in AF dysbiotic patients, with the exception of only one of the selected studies reporting an enrichment of this taxon correlated with AF [17]. However, this contrasting evidence is in line with numerous studies that highlighted both health-promoting and disease-triggering properties of *Prevotella* (pro-inflammatory effects). A potential explanation of this contradiction could be the huge strain variability, but also the lack of deep characterization of the genus itself [96].

Anyways, based on the GM alterations reported in the evaluated studies (Table 1), we are confident to conclude that dysbiosis is a clinical feature of atrial fibrillation, and monitoring certain taxa may provide therapeutic and preventive strategies. Furthermore, as investigated by Zuo and colleagues in 2019 and 2020, shifts in the patient enterotype occur to the same extent at different disease stages and tend to start early, thus strengthening the hypothesis of a GM-based strategy [8,17].

The cardiovascular effects of dysbiosis are mediated by bacterial metabolites, which can activate harmful pathways within the gut. Once in the bloodstream, they exert their effect on distant organs.

One of the most well-studied metabolites involved in atrial fibrillation is TMAO, which derives from the bacterial conversion of different dietary nutrients into trimethylamine (TMA). TMA is then released in the systemic circulation and reaches the liver, where it is oxidized to TMAO, the effector of inflammation and hypertrophy in the myocardium [97]. Experimental evidence supports a direct profibrotic role of TMAO, as demonstrated in a murine model where TMAO exposure promoted cardiac fibroblast proliferation, migration, and collagen deposition in a dose-dependent manner, mediated by activation of the TGF-β/Smad signaling pathway.

In an observational cross-sectional study from 2020, Zuo and colleagues revealed an enrichment of microbial genes implicated in the synthesis of TMA/TMAO in the microbiota of AF patients. Three genera, namely *Escherichia*, *Klebsiella*, and *Citrobacter*, were identified as taxa strongly associated with these genes, thus enriched in the gut of AF patients [15]. Recently, Paulina Hernández-Ruiz and colleagues, focusing on the association of oral microbiome and human myocardial infarction (MI), documented a positive and significant correlation of TMAO levels with Porphyromonas [98].

The TMAO role in AF was investigated in a canine model: this metabolite was injected into atrial ganglionated plexi to evaluate the effect on AF inducibility and progression. The results showed an increased atrial electrophysiological instability correlated with upregulation of pro-inflammatory cytokines and atrial remodeling, such as IL-1β, TNF-α, and IL-6 via NLRP3 inflammasome [23,99]. These findings suggested a potential TMAO role as a biomarker for cardiovascular diseases [100].

Two additional effectors in AF are LPS and bile acids, which are usually correlated with “leaky gut” and chronic inflammation and with atrial structural remodeling, respectively [101]. As reported in Table 1, only one study focused on the measurement of LPS levels, correlating them to age. Firstly, Zhang and colleagues compared aged rats with young ones, finding that aged animals were more susceptible to AF and displayed an altered GM profile. To confirm the hypothesis that aged microbiota may have an impact on AF development, they performed FMT between old and young rats and observed that the aged microbiota transplantation enhanced intestinal permeability and increased circulating LPS, triggering atrial TLR4/NF-κB signaling and NLRP3 inflammasome assembly in atrial tissues of young individuals. These findings were partially corroborated by the analysis of plasma samples from human donors and supported the correlation between aging, GM, metabolic changes, and AF [19]. The dysbiosis-related increase in LPS levels in the elderly is also supported by other studies about age-associated cognitive dysfunction and atherosclerosis, confirming a possible role of GM alterations in the onset of harmful inflammatory responses [102,103]. Similarly, in two studies, an increase in chenodeoxycholic acid was associated with AF [10,17]. Chenodeoxycholic acid is a primary bile acid that probably causes myocyte apoptosis, thus contributing to structural remodeling, raising the risk of AF [104]

The altered GM composition in atrial fibrillation also correlates with decreased levels of SCFAs due to the lack of bacterial species that physiologically produce these metabolites, having anti-inflammatory properties [101]. L. Chen et al. and Zuo et al. reported a reduction in isobutyric acid, isovaleric acid, acetate, and butyrate in AF patients. Both authors speculated that the decreased SCFAs’ availability exacerbates dysbiosis, perpetuating the inflammatory loop [11,25]. On the other hand, the protective role of SCFAs in AF was proved by Shi and co-workers, who proposed a rat model of atrial fibrillation, correlated with dysbiosis, induced by the drug Ibrutinib. When rats were supplemented with butyrate and the probiotic *Lactobacillus gasseri*, the GM balance was restored, and the fibrotic phenotype was ameliorated by suppressing ROS generation and preventing cardiomyocyte apoptosis [22]. In addition, other protective metabolites, such as α-linoleic acid, were reported to be negatively correlated with *Eubacterium* and *Blautia*, two of the genera enriched in AF patients [20]. The decrease in this fatty acid may exacerbate dysbiotic features because it is a positive modulator of the microbiota, also effective in alleviating gut inflammation [105]. Finally, regarding SCFA profiles, a recent study documented that healthy subjects displayed a higher abundance of isovaleric acid, whereas isobutyric and 2-methylbutyric acids were significantly higher in patients with ST-segment elevation myocardial infarction (STEMI) [106].

Based on the findings of the studies analyzed in this review, it can be stated that gut dysbiosis is surely a critical feature in atrial fibrillation, representing, at the same time, a risk and a driving factor of the disease. The enrichment in the relative abundance of specific genera could be a potential biomarker, as well as a target for AF modulation. Therefore, all the microbiota metabolites, which play a key role in the bacterial crosstalk and in the interaction with the host, are also gaining attention as predictive indicators. Taken together, all these factors may soon contribute to better management of the disease; however, it is worth noting that there are still some gaps that have to be addressed. Considering some contradictory results when focusing on the relative abundance of certain genera in AF patients, efforts are needed to characterize these alterations at the species level, since this approach will surely open up a different perspective in AF prevention and/or treatment. Moreover, further research should acknowledge the role of fungi and viruses because it cannot be excluded that they contribute to the complex interplay of the human microbiota, thus to the AF-correlated dysbiosis. Finally, the selection of innovative biomarkers, such as additional byproducts released or microbial extracellular vesicles-associated/delivered, should be detected for the prevention of AF, once their role in disease development is understood.

### 5.2. Limitations of the Studies

It is important to note that most of the original articles constituting the “core” of this review are observational studies having intrinsic limitations. The first aspect that has to be taken into account, according to the majority of the authors, is the enrolled population: a common issue was the relatively small sample size and, in some cases, the selection of a specific ethnicity [3,14]. Thus, to ensure representative findings, a higher number of subjects must be included, and the influence of genetic factors needs to be addressed. Moreover, additional confounding factors can be listed: dietary information, drugs or probiotics administration, and interaction between the patient’s diseases [11,16,24]. In addition, fecal or plasma sampling should be repeated at different time points, which is of particular importance when monitoring the circulant metabolites in AF patients. Finally, it has to be pointed out that the findings of observational studies are associations rather than casual relationship and must be confirmed by randomized controlled trials and other experimental studies focusing on the deepening of the mechanistic model describing the dysbiosis–AF correlation.

## 6. Conclusions and Future Perspectives

In conclusion, it can be stated that the gut microbiota and its metabolites are becoming recognized as biomarkers in AF. Alterations, such as the increase in *Bacteroides* and the decrease in *Faecalibacterium* or variations in TMAO and SCFAs, could help to identify or predict at-risk patients. Disruption of intestinal barrier integrity facilitates the translocation of LPS and other endotoxins, amplifying systemic inflammatory responses and promoting fibrotic remodeling and electrical instability. Specific microbial taxa may promote these pathogenic processes by degrading cardioprotective metabolites or impairing the intestinal mucus layer. On the other hand, a high-fiber and plant-based diet, together with butyrate supplements or specific probiotics, may help restore gut eubiosis and decrease inflammation. From a clinical perspective, the microbial and metabolic profiles represent a promising strategy since they could be used as biomarkers for early diagnosis and prevention of AF.

## Figures and Tables

**Table 1:** Gut microbiota alterations in atrial fibrillation in different studies.

Ref.	Study Type	Population	Gut Microbiota Alterations	Key Microbial Genes and Metabolites Identified	Proposed Mechanism	Clinical Implications
[8]	Observational case–control study	- 12 patients psAF < 12 months - 8 patients psAF > 12 months, - 20 CTRL	- Shift from *Prevotella* → *Bacteroides* - ↑ *Bacteroides*, *Ruminococcus* ↓ *Faecalibacterium praustnitzii* in long-duration AF - ↓ *Butyricicoccus*, *Paraprevotella*, and ↑ *Blautia, Dorea*, and *Coprococcus* in psAF of >12 - ↑ *Thermosinus*, *Anaeroarcus*, *Clostridium bolteae*, and *Enterococcus faecium* in psAF of >12 months - ↑ *Faecalibacterium* and *Corynebacterium* in psAF of <12 months	- KEGG modules: ↓ aminoacyl-tRNA biosynthesis, citric acid cycle, and iron complex transport in psAF patients - Metabolites: ↑ stearamide, octadecanedioic acid, and lysophosphatidylcholine in psAF patients compared to controls - ↓ oleic acid, choline, and some amino acids in psAF patients compared to controls	- Gut dysbiosis disrupts the balance between cardio-protective metabolites and harmful compounds - Dysbiosis is a clinical feature starting from the early stage of AF	- Cholin, niacin, and oleic acid could be used as biomarkers or therapeutic targets - The identification of early alterations, thus an earlier intervention, could prevent atrial fibrosis
[17]	Observational cross-sectional comparative study	- 50 nonvalvular AF patients (30 PAF, 20 psAF - 50 CTRLs	- ↑ *Bacteroides*, *Prevotella*, and *Faecalibacterium* in both PAF and psAF patients	- KEGG modules: ↓ citric acid cycle, iron complex transport system, aminoacyl-tRNA biosynthesis; ↑ fatty acids biosynthesis and pyrimidine deoxyribonucleotide biosynthesis in psAF compared to PAF patients - Metabolites: ↑ chenodeoxycholic acid, stearamide, lysophosphatidylcholine, and choline in AF - ↓ ∝-linoleic acid, niacin, indole, and phosphohydroxypyruvic acid in AF patients	- Alterations of gut microbiota appear already in PAF - Dysbiosis could be an initiating factor of AF - The production of altered metabolites leads to atrial remodeling and disturbed electrical signals in the heart tissues	Targeting dysbiosis and its metabolic pathways could be an effective strategy for AF prevention and/or treatment
[20]	Observational cohort study with Mendelian randomization analysis	- 40 patients with non-valvular AF undergoing radiofrequency catheter ablation (RAF group vs. non-RAF group) - 50 non-AF controls	- ↓ *Faecalibacterium* in AF patients, especially in RAF ones - ↑ pro-inflammatory genera such as *Ruminococcus*, *Eubacterium*, *Blautia*, *Dorea*, and *Dialister* in non-RAF patients, more pronounced in RAF ones - ↑ *Methanobrevibacter smithii* and *M. cruvatus* in RAF patients - ↓ *Faecalibacterium* sp. CAG:82, *Candidatus* sp., *Phycomycetaceae*, and *Bacteroides* bacterium RBG_13_43_22 in RAF	- ↑ LysoPC(15:0), lysoPE(0:0/16:0), chenodeoxycholic acid and sebacic acid; - ↓ LysoPE(0:0/20:0), corticosterone, α-linolenic acid, and uracil in AF - ↓ 7-Methylguanine and palmitoleic acid in RAF compared to non-RAF patients	- Gut dysbiosis could be associated with RAF after catheter ablation	Based on the gut microbiota profiling of RAF, non-RAF, and control samples, a Taxonomic Score can be introduced, which may represent a predictive strategy for recurrent AF
[9]	Observational cross-sectional study	- 92 patients with AF - 42 healthy controls - Murine atrial myocytes (HL-1 cells)	*ND*	- ↑ plasma levels of PAGln in AF patients - ↑ porA gene abundance in AF patients (not statistically significant)	- High PAGln levels directly correlate with left atrial diameters - PAGln induces in vitro apoptosis and ROS production, leading to a decrease in cell viability	- PAGln may be used as a biomarker aiding early diagnosis of AF
[10]	Observational cross-sectional study	- 50 patients with nonvalvular AF - 50 controls	- AF patients display a Bacteroides-dominated enterotype - ↑ *Ruminococcus*, *Streptococcus*, *Enterococcus*, *Blautia*, *Dorea*, *Veillonella*, and *Coprobacillus* in AF patients - ↑ *Escherichia coli*, *Eubacterium rectale*, *Bifidobacterium longum*, and *Collinsella aerofaciens* in AF patients - ↓ *Faecalibacterium*, *Prevotella*, *Alistipes*, *Oscillibacter*, and *Sutterella* in AF patients - ↓ *Faecalibacterium prausnitzii* in AF patients	- KEGG modules: ↓ fatty acids biosynthesis, aminoacyl-tRNA biosynthesis, citrate cycle, iron complex transport system, nucleotide sugar biosynthesis, and glycolysis i; ↑ histidine biosynthesis, heme biosynthesis, and pentose phosphate pathway in AF patients - EggNOG orthologs: ↓ DNA replication, cell wall/membrane biogenesis; ↑ carbohydrate transport and metabolism, signal transduction in AF patients. Metabolites: - ↑ chenodeoxycholic acid, lysophosphatidylcholine, and indole; - ↓ cholic acid, oleic acid, linoleic acid, alpha-linolenic acid, and several amino acids in AF patients	- The overgrowth of pro-inflammatory bacteria and the depletion of anti-inflammatory ones reduce the release of cardio-protective compounds, inducing, at the same time, the production of metabolites that participate in inflammation, oxidative stress, and atrial remodeling	- The microbiota-dependent discrimination model based on CAGs may have diagnostic potential - Targeting microbiota composition could provide novel strategies for AF treatment or prevention
[3]	Observational cohort study with Mendelian randomization analysis	- Dutch Microbiome Project: 7738 individuals - HUNT, deCODE, MGI, DiscovEHR, UK Biobank, and AFGen Consortium: 60,620 AF cases and 970,216 controls - FinnGen Cohort: 40,594 AF cases and 168,000 controls - UK Biobank: 430,000 participants	- Genus *Holdemania* and species *Eubacterium ramulus* are associated with increased risk of AF	*ND*	- *Eubacterium ramulus* degrades flavonoids, which are cardioprotective compounds, reducing their bioavailability: the reduction in flavonoids enhances coronary artery disease, which mediates AF - Genus *Holdemania* is involved in mucin degradation, which can promote intestinal barrier damage, triggering inflammation - Genus *Holdemania* is positively associated with increased BMI, which is known to be a risk factor for AF	Characterization of the gut microbiota could become a valuable tool for risk stratification or part of predictive models integrating genetic and clinical factors related to AF
[11]	Observational cross-sectional study	- 30 patients with AF - 30 patients with SR (control group)	- ↓ phylogenetic diversity in AF patients - ↓ Bifidobacteriales, Actinobacteria, and *Dialister* in AF patients - ↑ Pasteurellaceae, Fusobacteriaceae, and Lactobacillaceae in AF patients	- Metabolites: - ↓ Isobutyric acid and isovaleric acid; ↓ oxidative phosphorylation, glycine, serine, and threonine metabolism, and novobiocin biosynthesis in AF patients - ↓ N-acetyl-γ-glutamyl-phosphate reductase; - ↑ ATP-binding cassette in the AF group	- Altered levels of SCFAs within the gut are correlated to gut dysbiosis and contribute to exacerbating this condition: an increase in pro-inflammatory taxa	- SCFAs profile may be a biomarker for AF risk - Gut microbiota modulation and SCFAs levels restoration may be included as novel therapeutic strategies - Early gut microbiota profiling and SCFAs identification may represent a preventive strategy
[2]	Observational cohort study with Mendelian randomization analysis	- European individuals from multiple large-scale GWAS: 60,620 AF cases and 970,216 controls	- Taxa that tend to genetically reduce the risk of AF: Lentisphaeria, Bifidobacteriaceae, *Anaerostipes*, *Howardella*, *Intestinibacter*, Lachnospiraceae NK4A136 group, *Odoribacter*, *Ruminococcus gnavus* group, Lentisphaerae - Taxa associated with increased AF risk: Lachnospiraceae UCG008, Rickenellaceae RC9 gut group and *Streptococcus*	- Protective cytokines: Fms-related tyrosine kinase 3 ligand, IL-6, IL-7, leukemia inhibitory factor receptor, sulfotransferase 1A1, TNF ligand (superfamily member 12) and CD40L receptor - Risk-enhancing cytokines: FGF5, IL-2R subunit β and TNF	- Gut dysbiosis leads to the disruption of intestinal barrier integrity: microbial metabolites can more easily enter the circulation and trigger inflammation, which contributes to atrial fibrosis and remodeling	- Gut microbiota profiling and inflammatory cytokine levels could be considered biomarkers for AF risk assessment - Preventive and therapeutic strategies may be based on gut microbiota modulation and anti-inflammatory treatments
[12]	Observational cross-sectional study	- 50 patients with AF (32 with high CHA2DS2-VASc score and 18 with low CHA2DS2-VASc score)	- ↑ *Bacteroides* in patients with high CHA2DS2-VASc score - ↓ *Prevotella* and *Eubacterium rectale* in patients with high CHA2DS2-VASc score	84 KEGG Orthologs were differentially enriched between the two groups: - ↑ 62 KOs (metabolism of SCFAs, degradation of histidine, serine, 4-methylcatechol, nitrotoluene, and aminobenzoate, and activation of bacterial urease) in high CHA2DS2-VASc patients - ↑ 22 KOs (ascorbate and histidine biosynthesis) in low CHA2DS2-VASc patients	- ↓ SCFA-producing bacteria lead to a more inflammatory environment, promoting processes that increase the risk of thromboembolism - Microbial metabolic changes can influence inflammatory responses, oxidative stress, and endothelial function, conditions that promote thrombosis and increase cardiovascular risk.	Gut microbiota profiling could have the potential to become an additional marker of thromboembolic risk in patients with AF
[19]	Observational cross-sectional and in vivo study	- Old rats (22–24 months) - Young rats (2–3 months) - Plasma LPS/FA prevalence 1152 patients - OGTT: 12,012 human donors - Atrial samples: 18 patients undergoing cardiac surgery	- ↑ Proteobacteria, ↑ Spirochaetae, ↑ Bacteroidetes/Firmicutes ratio, and ↓ Verrucomicrobica in aged rats - ↓ Bacteroidaceae in aged rats	- ↑ LPS in elderly AF patients - ↑ LPS in aged rats and young rats receiving FMT from aged rats - ↑ intestinal permeability in aged rats correlated with ↑ circulating LPS	- Aging leads to intestinal dysbiosis and an alteration of the intestinal barrier - High levels of LPS in combination with high glucose levels appear to be closely related to an increased risk of developing AF - High levels of LPS and glucose may be correlated with the activation of the NLRP3-inflammasome	The gut microbiota is a potential therapeutic target for age-related AF
[18]	Observational cross-sectional comparative study	- 27 HTN patients with AF (HTN-AF) - 27 non-AF HTN patients	- ↑ enterotype *Bacteroides* and ↑ *Ruminococcus*, *Streptoccus*, *Veillonella*, *Dorea*, and *Enterococcus* in HTN-AF patients - ↑ enterotype *Prevotella* in HTN patients	56 KEGG metabolic pathways were significantly different between the two groups, all related to metabolism. - Metabolic pathways: ↑ linoleic acid metabolism, arachidonic acid metabolism, secondary bile acid biosynthesis in HTN-AF patients - ↑ Flavonoid biosynthesis, stilbenoid, diarylheptanoid, and gingerol biosynthesis; α-linolenic acid metabolism; primary bile acid biosynthesis in non-AF HTN patients	Intestinal dysbiosis and an alteration in related metabolites may contribute to the development of AF in hypertensive patients	Microbial and metabolic profiles may represent predictive tools for the screening of patients at higher risk of developing AF
[4]	Observational cross-sectional and short-term longitudinal study	- 36 patients with non-valvular AF - 30 healthy controls, matched for age, sex, BMI, hypertension, and diabetes. - 18 patients followed up before and after ablation	- ↑ *Bacteroides*, *Klebsiella*, *Megamonas*, *Methylobacterium-Methylorubrum*, *Parabacteroides*, *Streptococcus*, *Weissella*, *Alistipes*, *Haemophilus*, and *Enterococcus* in AF patients	- Metabolites: ↓ Flavin adenine dinucleotide (FAD); Riboflavin-5-phosphate (active vitamin B2); Inosine; Dehydroepiandrosteron (DHEA); Oestradiol; Caffeine; Salicylic acid; Ascorbic acid (vitamin C); Eicosapentaenoic acid (EPA); Oleanolic acid; ↑ N-acetylmethionine and β-hydroxy-β-methylbutyric acid in AF patients. - ↑ Citrulline6-hydroxymelatonin; Homovanillic acid; ↓ Flavin adenine din Pelargonidin, α-Linolenic acid, Linolelaidic acid, Oleanolic acid, Phosphatidylcholine (PC) in AF patients after ablation	- LPS and pro-thrombotic metabolites (e.g., TMAO) are linked to AF development - ↑ Beneficial SCFAs-producer contribute to cardiovascular protection - Catheter ablation is associated with partially restored eubiosis and improved metabolic profile	Gut microbiota profiling and analysis of metabolites may be used as potential biomarkers in AF
[21]	Prospective observational cohort study	People aged between 25 and 74 from six geographical regions are invited to participate in the FINRISK 2002 study: - 6763 individuals ∘ 116 patients with prevalent AF ∘ 6.647 individuals non-AF at the baseline (539 developed AF during the follow-up = incident AF) :	- ↑ *Enorma*, *Eisenbergiella*, *Enterobacter*, and *Kluyvera*; - ↓ *Bacteroides*, *Bifidobacterium*, *Holdemanella*, *Parabacteroides*, and *Turicibacter* are prevalent in AF patients - ↑ *Bifidobacterium*, *Enorma*, and *Lactococcus*; - ↓ *Tyzzerella* and *Hungatella* in incident AF patients - *Bifidobacterium*, *Enorma*, and *Lactococcus* were more prevalent in subjects who subsequently developed AF, while *Tyzzerella* and *Hungatella* were less prevalent	*ND*	In AF patients, there is a shift in the microbiota composition that is close to that in hypertension and heart failure: this suggests that there could be a similar pathophysiology	Gut microbiota may potentially have a predictive role in the risk of developing AF
[13]	Observational cross-sectional study	- 50 AF patients (non-valvular) and 50 healthy controls → metagenomic analysis - 23 AF patients and 23 controls → targeted metabolomics (UPLC-MS/MS) - 36 AF patients and 24 controls → plasma FGF19 measurement - HL-1 cells and Caco-2 cells → in vitro experiments	↓ bacteria involved in the biotransformation of bile acids: *Faecalibacterium*, *Roseburia*, *Dialister*, *Butyricicoccus*, *Prevotella*, *Eubacterium*, and *Blautia*.	In patients with AF ↓, enzymes encoded by the bai operon are involved in the transformation of bile acids (*7α-HSDH*; *7β-HSDH*; *baiA*, *baiA2*, *baiH*, *baiCD*, and *baiN*)	Intestinal dysbiosis leads to impaired conversion of primary bile acids to secondary bile acids and decreased plasma FGF19, resulting in increased lipid accumulation and atrial cardiomyocyte dysfunction	The microbiota-bile acids-FGF19 axis is a hypothetical biomarker of AF risk
[14]	Observational cross-sectional study	- 1475 participants (40–75 years old): followed up every 3 years - 199 (52–83 years old): replication cohort	- ↑ Burkholderiales and Alcaligenaceae in AF patients - ↓ *Lachnobacterium*, *Bacteroides coprophilus*, Barnesiellaceae, an undefined genus in the family Veillonellaceae, and *Mitsuokella* in AF patients	*ND*	Host genetics may influence gut microbiota composition, inducing a shift similar to that of AF patients	- Gut microbiota could be a potential biomarker or target for AF risk modulation - Host genetics may have an impact on gut microbiota modulation, but these findings are hypothesis-generating and require validation in clinical cohorts
[22]	Preclinical experimental study	- 8-week-old male Wistar rats (200–240 g): Ibrutinib treatment and burst pacing to induce AF - Rat cardiomyocytes and Caco-2 cells → in vitro butyrate and *L. gasseri* treatment	- ↑ Bacteroidota; - ↓ *Spirochaeta* and *Lactobacillus gasseri* abundance in Ibrutinib-treated rats	- ↓ Butyrate in Ibrutinib-treated rats - ↑ Butyrate in Ibrutinib- and *L. gasseri*-treated rats - ↓ Acetate, propionate, and NF-κB pathway in Ibrutinib-treated rats - ↓ ROS, IL-6, and TNF-α and ↑ Nrf2 in cells treated with butyrate or *L. gasseri*	- The inflammation and dysbiosis induced after Ibrutinib treatment led to AF development - Butyrate and *L. gasseri* treatment can restore the gut microbiota balance	Supplementation of butyrate and *L. gasseri* could help restore the gut microbiota for the prevention and management of ibrutinib-associated AF
[23]	Preclinical experimental study	- 21 mongrel dogs weighing 20 to 24 kg were used in this experiment: TMAO injection to induce AF	*ND*	*ND*	- TMAO injected into atrial GP increases autonomic nervous system activity, enhancing ARGP function and neural activity: this leads to shortening atrial ERP, increasing atrial electrophysiological instability - TMAO exacerbates electrical remodeling in RAP-induced AF by activating pro-inflammatory p65 NF-κB signaling pathway - TMAO upregulates inflammatory cytokines contributing to AF progression	Targeting TMAO or its downstream signaling pathways could provide novel therapeutic approaches for AF prevention or treatment
[15]	Observational cross-sectional study	- 50 patients with nonvalvular AF - 50 non-AF controls	↑ *Escherichia*, *Klebsiella*, *Kluyvera*, and *Citrobacter* (associated with TMAO production) in AF patients	↑ Genes encoding TMA-synthesis enzymes (CutC, CntA, GrdH, TorA) in AF patients compared with controls	- Specific taxa, enriched in AF patients, harbor TMA-synthesis genes converting dietary choline, carnitine, and betaine into TMA, which is then metabolized into TMAO - ↑ TMAO levels may activate the cardiac autonomic nervous system, promoting atrial electrophysiological remodeling, and inducing inflammation, thereby increasing AF susceptibility and progression	Targeting TMA-synthesis pathways and gut dysbiosis could serve as therapeutic strategies for AF
[24]	Prospective observational study	- 34 AF patients from the hospital - 66 controls	- ↑ enterotype *Ruminococcus*-dominant; ↓ enterotype *Prevotella*-dominant; - ↑ *Parabacteroides*, *Lachnoclostridium*, *Streptococcus*, *Alistipes*, *Dorea*, *Butyricimonas*; - ↓ Enterobacter in AF patients	*ND*	Enrichment of *Streptococcus*, *Lachnoclostridium*, *Parabacteroides*, *Alistipes*, *and Dorea* could be related to increased TMAO and LPS levels that may be the trigger for oxidative stress and atrial electrophysiology instability, leading to increased risk of AF	- The gut microbial dysbiosis could be a risk factor for AF development - Metabolites produced by certain taxa may serve as potential biomarkers or therapeutic targets
[25]	Observational cross-sectional study combined with in vitro and in vivo experiments	- 24 AF patients - 24 healthy controls - Six- to eight-week-old wild-type male C57BL/6 mice - HL-1 cells	- Progressive decrease in total SCFA levels in human fecal samples Mouse fecal samples: Low-fiber diet: - ↑ Gut microbial diversity - ↑ *Ruminococcus* (pro-inflammatory genus) - ↓ *Bacteroides* (SCFA-producing genus) High-fiber diet: - ↑ *Akkermansia* (beneficial, supports SCFA producers)	- ↓ Acetate, butyrate in AF patients and low-fiber diet mice (restored with SCFAs supplementation) - ↓ Propionate in low-fiber diet mice (restored with SCFAs supplementation) - SCFAs → ↓ GPR43 → ↓ NLRP3 → ↓ inflammation in HL-1 cell line	- Commensal bacteria are essential for fiber fermentation, which leads to SCFAs production - SCFAs negatively modulate the inflammasome activation, preventing the effect on atrial remodeling	- Monitoring SCFAs levels may be a strategy to detect AF risk - A high-fiber diet may help prevent AF or slow its progression - SCFAs-based therapies may help reduce inflammation
[16]	Observational cross-sectional study	- 53 AF patients - 29 healthy patients	- AF groups divided by CHA2DS2-VASc: AF-0, AF-1, AF-2 vs. Healthy (H). - AF-2: ↑ Firmicutes - Healthy: ↑ Chloroflexi, Sphingobacteriia, Flavobacteriia, Alphaproteobacteria. Genus-level differences: - ↑ *Sphingobacterium* (Healthy) - ↑ *Clostridium* sensu stricto1(AF-2) - ↑ *Dialister*, *Allisonella* (AF-1) - ↑ *Prevotella*-9 (AF-0) - AF subgroups with higher CHA2DS2-VASc scores showed ↑ Firmicutes and ↓ *Sphingobacterium*, Chloroflexi	*ND*	- Elevated CRP levels are associated with AF. - Several taxa (Acidobacteria, Rhodospirillales, Moraxellaceae, Nocardiaceae) were negatively correlated with CRP levels - Gut dysbiosis → ↑ TMAO production → activation of pro-inflammatory signaling → ↑ CRP → vascular inflammation and myocardial remodeling → onset or recurrence of AF - Heart failure → intestinal congestion and microcirculatory disturbance → ↑ intestinal permeability and systemic inflammation → worsened cardiac function. - Conversely, beneficial bacteria (e.g., Sphingobacteriaceae) may help maintain better cardiac performance. - Altered gut flora → ↑ TMAO → enhanced oxidative stress and inflammation → myocardial fibrosis → atrial fibrillation. - Gut dysbiosis may both reflect and contribute to cardiovascular risk factors included in the CHA2DS2-VASc score. - Firmicutes are known to be correlated with hypertension and atherosclerosis.	- Specific microbial patterns (e.g., Firmicutes/Bacteroidetes ratio, *Prevotella*-9, Sphingobacteriaceae) could serve as non-invasive biomarkers of inflammation or thromboembolic risk in AF patients. - Modulating gut flora (through diet, probiotics, or reducing TMAO) may become a novel therapeutic strategy to prevent or manage AF, especially in high-risk patients. - Integration of microbial and metabolic markers with the CHA2DS2-VASc score could improve cardiovascular risk prediction. - Recognizing the bidirectional relationship between gut dysbiosis and cardiac dysfunction highlights the potential for a holistic management of AF patients.

↑ = increase; ↓ = decrease; ND = Not Detected. Abbreviations: psAF, persistent Atrial Fibrillation; PAF, Paroxysmal Atrial Fibrillation; LC-MS, Liquid Chromatography-Mass Spectrometry; RAF, Atrial Fibrillation Recurrence; CAGs, Co-Abundance Gene Groups; PAGln, Phenylacetylglutamine; BMI, Body Mass Index; SR, Sinus Rhythm; SCFAs, Short-Chain Fatty Acids; GWAS, Genome-Wide Association Studies; IL, Interleukin; TNF, Tumor Necrosis Factor; FGF, Fibroblast Growth Factor; UPLC-MS/MS, Ultra-Performance Liquid Chromatography-tandem Mass Spectrometry; FMT, Fecal Microbiota Transplantation; HE, Haematoxylin and Eosin; TMAO, Trimethylamine N-Oxide; IS, Indoxyl Sulfate; GP, Atrial Ganglionated Plexi; ARGP, Anterior Right GP; ERP, Effective Refractory Period; RAP, Rapid Atrial Pacing; CutC, Choline-TMA Lyase; CntA, Carnitine Monooxygenase; GrdH, Glycine Betaine Reductase; TorA, TMAO Reductase; CHA_2_DS_2_-VASc = Congestive Heart Failure Score (C); Hypertension (H); Age ≥ 75 years (A_2_); Diabetes Mellitus (D); Stoke/Transient Ischemic Attack (TIA) or Thromboembolism (S_2_); Vascular Disease (V); Age (A); Sex Category (Sc); HNT, Hypertensive Patients Without Atrial Fibrillation; AF-HNT, Hypertensive Patients With Atrial Fibrillation; KOs, KEGG Orthology-Based Scoring; OGTT, Oral Glucose Tolerance Test; CRP, C-Reactive Protein.

## Data Availability

No new data were created or analyzed in this study. Data sharing is not applicable to this article.
